# Development and validation of a new RP-HPLC method for organic explosive compounds

**DOI:** 10.55730/1300-0527.3380

**Published:** 2022-02-25

**Authors:** Salih Murat ÜNSAL, Emre ERKAN

**Affiliations:** Gendarmerie Criminal Department, Ankara, Turkey

**Keywords:** Optimization, organic explosive, RP-HPLC, stationary phase, validation

## Abstract

A new RP-HPLC method was developed and validated to achieve the separation and quantification of the organic explosive compounds such as pentaerythritol tetranitrate (PETN), 1-methyl-2,4,6-trinitro toluen (TNT), picric acid, 1,3,5,7-tetrazocine (HMX), cyclorimethylenetrinitramine (RDX), 2,4,6 Tri nitro phenyl methyl nitramine (Tetryl), 1-methyl-2,4-dinitro toluen (DNT), ethylene glycol dinitrate (EGDN), and trinitroglycerine (TNG) in this study. The mobile phase composition (IPA percentage in water) and the flow rate was optimized for the separation of the organic explosive compounds. Theoretical plate number (N), capacity factor (k′), resolution (Rs) were used to determine the optimum chromatographic conditions. The most favorable conditions were detected as 22% IPA in water, a flow rate of 1.7 mL/min. Under optimum chromatographic conditions, separation was completed within 18 min. The linear ranges were 6.5–100 and 10–0.625 mg/L (R^2^ = 0.998–0.999) for investigated explosives. Mean recoveries were found to be in the range of 95.3%–103.3%. LOD and LOQ were ranged between 0.09–1.32 mg/L and 0.31–4.42 mg/L, respectively, for investigated explosives. The obtained results showed that the new method was very suitable for the separation of the resolution of organic explosive compounds with C18 columns. TNT and RDX seized from terrorists were found to be in 372.99 mg/L and 79.55 mg/L, respectively.

## 1. Introduction

Explosives, which are mostly used in terrorist incidents, and their analysis are of great importance in the field of forensic sciences. Analysis of explosives in a result-oriented manner is important in terms of clarifying the investigation and prosecuting suspicious persons. Analyses of the explosive materials are carried out for the detection and examination of explosive material residues on the findings after the explosion and before the explosion in their original form [1, 2].

Organic materials such as pentaerythritol tetranitrate (PETN), 1-methyl-2,4,6-trinitro toluen (TNT), picric acid, 1,3,5,7-tetrazocine (HMX), cyclorimethylenetrinitramine (RDX), 2,4,6 Tri nitro phenyl methyl nitramine (Tetryl), 1-methyl-2,4-dinitro toluen (DNT), ethylene glycol dinitrate (EGDN), and trinitroglycerine (TNG) are used worldwide as explosives by primary military and terrorist elements [1–3]. The main constituents of these materials are nitramines, nitrate esters, and nitroaromatics (NACs) and are highly energetic [4,5]. Almost all organic explosives contain nitrogenous structures and N_2_ is released as a result of explosion [6]. According to their strength, explosives are divided into two main classes as strong and weak. While TNT, PETN, and RDX, which are among the powerful explosives, have crushing, crushing and disintegrating effects on their targets, Tetryl and TNG are noninitiating and relatively insensitive to heat, shock, and friction. TNT, PETN, and RDX are very sensitive to heat, shock, and friction as well as showing strong effects [4]. In order to create a greater effect, energy, and a high detonation velocity, explosive materials are used alone as well as in binary and multiple mixtures in improvised explosive devices (IEDs) from terrorist attacks [2, 7–10].

The rapid analytical techniques such as nonaqueous titration [11], colorimetric [12], fluorometric [13], spectrophotometric [14,15], chromatographic [16–19], electrophoresis [16], raman [20], and electrochemical [21–23] methods are developed and used for individual and simultaneous determination of organic explosive materials including PETN, TNT, picric acid, HMX, RDX, Tetryl, DNT, EGDN, and TNG [24]. Reverse phase liquid chromatography is the most widely used for the identification and determination of these materials by using conventional C8 and C18 columns [25,26]. These compounds, which are thermally unstable and need to be derivatized for different chromatographic methods, can be simultaneously determined by liquid chromatography without any pretreatment [25–27].

The aim of this study was to investigate the efficiency of the new RP-HPLC method and its validation for organic explosive compounds separation in binary and multiple mixtures in improvised explosive devices. For this purpose, the effects of mobile phase composition (IPA percentage in water) and flow rate were investigated and validation parameters were successfully employed.

## 2. Materials and methods

### 2.1. Chemicals and reagents

TNG, EGDN, PETN standards were purchased from HPC (Cunnersdorf, Deutschland), HMX, TNT, RDX and Tetryl purchased from Ultra Scientific (North Kingstown, USA) and picric acid and DNT others were purchased from Sigma-Aldrich and Supelco (Germany). Other chemicals and solvents used in the analysis were chromatographic purity and purchased from Merck, Fluka, Sigma-Aldrich, Supelco, and LabScan companies. Standards and real explosive sample including TNT and RDX seized from terrorists were solved in water-ACN (60%–40%) and filtered by 0.45 μm PTFE syringe tip filter. The sample and standard mix solutions were directly injected into the HPLC system.

### 2.2. HPLC system and optimization of chromatographic conditions

Organic explosive compounds analysis was performed with a 1100 series Agilent Technologies (Agilent Technologies Inc., Wilmington, DE, USA) including a JP13211005 degasser, a G 1329B ALS automatic injector system, a G1311A model quaternary pump, a G1315B model diode array detector. Sample (10 μL) was directly injected into Eclipse XDB-C18 (5 μm–4,6 × 150mm) column. The temperature was at 25 °C. DAD detector set at 200 nm for PETN, DNT, HMX, RDX, EGDN, 210 nm for picric acid and TNG, 222 nm for TNT and Tetryl.

For optimum chromatographic conditions on Eclipse XDB-C18 (5 μm–4,6 × 150mm) column, mixture of isopropyl alcohol-water in different proportions for mobile phase and different flow rates were applied. The performance of HPLC methods was assessed by altered mobile phase and flow rate as shown in Table 1. Standards of 6.25 ppm for picric acid, HMX, RDX, TNT, Tetryl, PETN and 0.625 ppm for TNG, DNT, EGDN were used to determine the appropriate method. Chromatographic separation efficiencies were calculated using a capacity factor, k; selectivity, α; number of theoretical plates, N and resolution, Rs.

### 2.3. Method validation

A recovery test for accuracy, linear range, relative standard deviation (RSD%) for precision, correlation coefficients (R^2^) for linearity, the limit of detection (LOD), and limit of quantification (LOQ) for sensitivity were used and calculated for method validation. An organic explosive compounds mix standard was injected 10 times for validation. The mean, standard deviation, and R^2^ values were calculated with the help of the peak areas. A recovery test of the method was carried out by adding known amounts of organic explosive compounds (10 mg/L) to a sample blank that have not included these compounds. The LOD and LOQ of the method were calculated out [28].

## 3. Results and discussions

### 3.1. Optimization of chromatographic conditions

There are many HPLC methods available in the literature for the determination of explosives. However, most of these methods can simultaneously detect one or a few explosives [29, 30, 31]. For this purpose, a new method has been developed by changing the mobile phase composition (isopropyl alcohol-water) and flow rate in order to achieve better separation and simultaneous analysis of many explosives. The 8 different HPLC methods were used to separate explosives in different sources for optimization of chromatographic conditions. The k′, α, N, and Rs values calculated by using the obtained data from the analysis results were used in the evaluation of the chromatographic analyzes. The k′, the Rs value which has closest to 1.5 and the number of theoretical plates, which has the highest value, was used in the evaluation of the chromatographic separation methods. TNT and Tetryl, which have very close retention times, were taken especially into account to compare the resolution and chromatographic performance of the methods. The retention time of picric acid was taken into account to calculate the k′ value. The results of the chromatographic separation efficiency calculations of the used methods are given in Table 1.

IPA percentage and flow rate significantly affected the separation process of explosives. While higher k′ was calculated with the lowest IPA percentage in the mobile phase studies, the k′ value decreased in the highest IPA percentage. However, no significant change was observed in k′ values in flow rate changes. At the same time, there was no significant change in the α values in both different IPA percentages and flow rates.

Considering the theoretical plate results, it was observed that the number of plate decreased as the IPA ratio decreased and the flow rate increased, as in the k values. It was observed that the polarity changes of the mobile phase had a significant effect on the theoretical plate number. However, as the flow rate increases, it is thought that the stationary phase interactions with the analytes decreased.

It was observed that when the percentage of IPA was increased, the value of Rs also increased. Depending on the increase in polarity, the separation of TNT and Tetryl was very effective. The exact reason for the Rs value in method-1 cannot be fully explained. After determining the composition of the mobile phase, it was observed in the flow rate studies that the effect of the flow rate on the Rs value was not as much as the mobile phase. However, the flow rate was determined by considering the best separation in the shortest time and using the least chemical substance, which is the principle for chromatographic separations [28].

**Figure 1:**
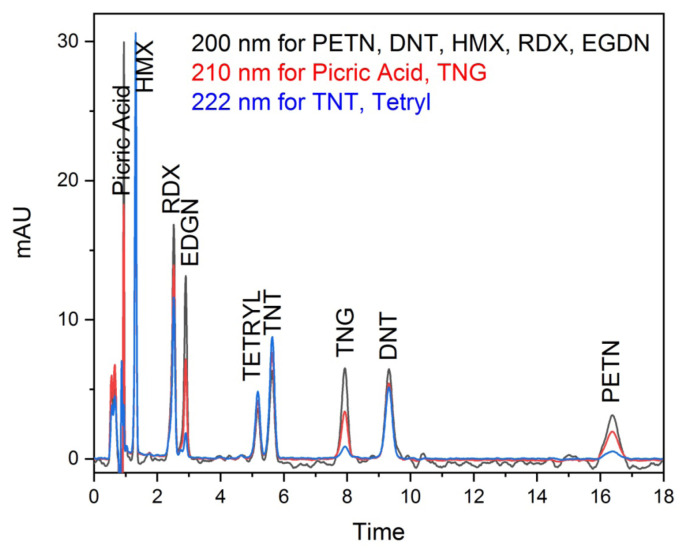
Chromatogram of the mix explosive standards.

Considering the calculated chromatographic yield results, Method-3 was found to be the most appropriate method for the simultaneous analysis of explosives. The chromatographic conditions were optimized for mobile phase composition of IPA percentage of 22 and flow rate of 1.7 mL/min. The chromatogram of the mixed explosives made under these conditions was shown in Figure 1. Organic explosive compounds analysis was achieved in 18 min.

### 3.2. Method validation and analysis of explosives in real sample

Validation is the statistical process applied to independently test and prove the suitability, precision, accuracy, and reproducibility of any method for the analysis of predetermined species in all analytical techniques [28]. For this propose, the new RP-HPLC method for organic explosive compounds was validated in terms of accuracy, linear range, relative standard deviation, correlation coefficients, the limit of detection and the limit of quantification at optimized conditions (Table 2). RSD values showing precision were calculated using the standard concentrations after 10 repetitive injections and were found in the range of 1.23–3.57. RSD values were calculated more than 2% for picric acid, EGDN, TNG and DNT. The R^2^ using the least-squares method for linearity of the method using the calibration curves obtained at ten different concentration levels of organic explosive compounds were calculated in the range of 0.998–0.999. Recovery tests for the accuracy of the developed method were found in the range of 95.3%–104.3%. The sensitivity of the new method was evaluated by calculating the limit of detection (LOD) and limit of quantification (LOQ) of organic explosive compounds. LOD and LOQ for organic explosive compounds were calculated in the range of 0.09–1.32 ppm and 0.31–4.42 ppm, respectively.

**Figure 2:**
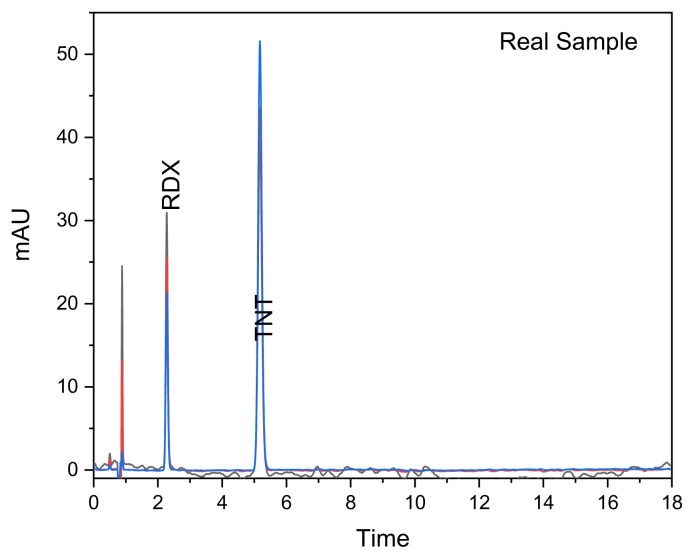
Chromatogram of the rea sample.

Although studies on organic explosives have been comprehensively searched in the literature, limited studies have been found. Among these studies, 8 analytes which are explosives and organic gunshot residues were determined by Şener, Anilanmert (31) et al. using LC-APCI-MS/MS. LOD and LOQ values were found in the range of 0.2 and 132.3ng/g and 0.3 and 355.0 ng/g, respectively. These values were lower than our study due to the MS-MS detection limit. However, recovery values were similar to ours. Real sample application of developed new RP-HPLC method for the simultaneous determination of organic explosive compounds was verified in the real explosive sample including TNT and RDX seized from terrorists (Figure 2). TNT and RDX seized from terrorists were found to be in 372.99 mg/L and 79.55 mg/L, respectively.

## 4. Conclusion

In conclusion, a new RP-HPLC method has been developed for the simultaneous separation of organic explosive compounds such as PETN, TNT, picric acid, HMX, RDX, Tetryl, DNT, EGDN, and TNG in the field of forensic sciences. New RP-HPLC method conditions including mobile phase composition (IPA percentage in water) and flow rate were optimized. For this purpose, chromatographic separation efficiencies were calculated by using a capacity factor, selectivity, number of theoretical plates, and resolution. The most suitable method for simultaneous analysis of explosives was determined to be 22% IPA and 1.7 mL/min flow rate. Validation for the new HPLC method was carried out and evaluated in terms of accuracy, linear range, relative standard deviation (RSD%) for precision, correlation coefficients (R^2^) for linearity, the limit of detection (LOD), and limit of quantification (LOQ). As a result, the present study showed that the new RP-HPLC method could be used for the separation, identification, and quantification of organic explosive compounds in forensic sciences.

## Figures and Tables

**Table 1:** Chromatographic separations conditions and yields for methods.

	IPA (%)	Water (%)	Flow rate (mL/min)	k′	α	N	Rs
**Method-1**	15	85	1.7	0.70	1.07	9610	1.46
**Method-2**	20	80	1.7	0.67	1.05	6135	0.91
***Method-3***	***22***	***78***	***1.7***	***0.67***	***1.09***	***5198***	***1.57***
**Method-4**	25	75	1.7	0.52	1.19	5088	2.89
**Method-5**	30	70	1.7	0.37	1.32	4067	3.85
**Method-6**	22	78	1.25	0.48	1.10	6908	1.85
**Method-7**	22	78	1.5	0.59	1.10	5978	1.67
**Method-8**	22	78	2	0.43	1.10	5172	1.49

**Table 2:** Validation results.

	Picric acid	HMX	RDX	EGDN	Tetryl	TNT	TNG	DNT	PETN
**%RSD**	2.39	1.89	1.23	3.57	1.46	1.43	2.78	3.28	1.35
**%Recovery**	103.3	101.6	100.9	104.3	95. 3	95.3	101.7	98.2	100.9
**LOD (mg/L)**	1.32	0.34	0.14	0.34	0.09	0.42	0.33	0.17	0.47
**LOQ (mg/L)**	4.42	1.14	0.47	1.14	0.31	1.39	1.10	0.56	1.58
**R** **^2^**	0.998	0.999	0.999	0.999	0.999	0.999	0.999	0.999	0.999
**Linear range (mg/L)**	6.5–100	6.5–100	6.5–100	10–0.625	6.5–100	6.5–100	10–0.625	10–0.625	6.5–100
**Intraday %RSD**	1.31	1.01	1.00	2.1	0.98	1.10	0.88	2.12	1.01
**Interday %RSD**	1.10	1.70	1.13	2.75	1.32	1.01	2.10	2.75	1.12
